# RNA-seq analysis of the *Rhizobium tropici* CIAT 899 transcriptome shows similarities in the activation patterns of symbiotic genes in the presence of apigenin and salt

**DOI:** 10.1186/s12864-016-2543-3

**Published:** 2016-03-08

**Authors:** Francisco Pérez-Montaño, Pablo del Cerro, Irene Jiménez-Guerrero, Francisco Javier López-Baena, Maria Teresa Cubo, Mariangela Hungria, Manuel Megías, Francisco Javier Ollero

**Affiliations:** Departamento de Microbiología, Facultad de Biología, Universidad de Sevilla, Avenida Reina Mercedes n° 6, 41012 Sevilla, Spain; Embrapa Soja, Londrina, Paraná Brazil

**Keywords:** RNA-seq, *Rhizobium tropici* CIAT 899, Nodulation, Nod factors, Lipochitooligosaccharides, Apigenin, Salt stress

## Abstract

**Background:**

*Rhizobium tropici* strain CIAT 899 establishes effective symbioses with several legume species, including *Phaseolus vulgaris* and *Leucaena leucocephala*. This bacterium synthesizes a large variety of nodulation factors in response to *nod*-gene inducing flavonoids and, surprisingly, also under salt stress conditions. The aim of this study was to identify differentially expressed genes in the presence of both inducer molecules, and analyze the promoter regions located upstream of these genes.

**Results:**

Results obtained by RNA-seq analyses of CIAT 899 induced with apigenin, a *nod* gene-inducing flavonoid for this strain, or salt allowed the identification of 19 and 790 differentially expressed genes, respectively. Fifteen of these genes were up-regulated in both conditions and were involved in the synthesis of both Nod factors and indole-3-acetic acid. Transcription of these genes was presumably activated through binding of at least one of the five NodD proteins present in this strain to specific *nod* box promoter sequences when the bacterium was induced by both apigenin and salt. Finally, under saline conditions, many other transcriptional responses were detected, including an increase in the transcription of genes involved in trehalose catabolism, chemotaxis and protein secretion, as well as ribosomal genes, and a decrease in the transcription of genes involved in transmembrane transport.

**Conclusions:**

To our knowledge this is the first time that a transcriptomic study shows that salt stress induces the expression of nodulation genes in the absence of flavonoids. Thus, in the presence of both nodulation inducer molecules, apigenin and salt, *R. tropici* CIAT 899 up-regulated the same set of symbiotic genes. It could be possible that the increases in the transcription levels of several genes related to nodulation under saline conditions could represent a strategy to establish symbiosis under abiotic stressing conditions.

**Electronic supplementary material:**

The online version of this article (doi:10.1186/s12864-016-2543-3) contains supplementary material, which is available to authorized users.

## Background

Rhizobia comprise a group of α- and β-proteobacteria known for their ability to establish symbioses with several leguminous species. The rhizobium-legume interaction, characterized by an exchange of signal molecules from both partners, culminates in the formation of specific structures, called nodules, where biological nitrogen fixation takes place [1–5]. This molecular dialogue begins with the exudation of flavonoids by the host legume roots that are recognized by a LysR-type transcriptional regulator in the bacterium, the NodD protein, which triggers the expression of the so-called nodulation (*nod*) genes by binding to specific sequences, *nod* boxes (NB), located upstream of these genes. Their cognate enzymes are implied in the production of lipochitooligosaccharides, also known as Nod factors (NF), which in turns induce the formation of root nodule primordia and play an essential role in the infection process. A part from flavonoids, other rhizobial *nod* gene inducers have been identified, such as betaines [6], but they are required at higher concentrations [7].

*Rhizobium tropici* CIAT 899 (hereafter CIAT 899) is a broad host-range rhizobial strain isolated from tropical acid soils of South America that effectively nodulates several legumes, including *Phaseolus vulgaris*, *Macroptilium atropurpureum*, and *Leucaena leucocephala* [8, 9]. Main characteristics of this strain includes its high tolerance to several environmental stresses such as high temperature, acidity or salinity and its capacity to producing a large variety of NF in the presence of inducer flavonoids, such as apigenin [8, 10, 11]. It is remarkable that under acidity or salt stress conditions the synthesis of NF in CIAT 899 is also induced, resulting in increased diversity and concentration of these molecules in comparison to non-stressing conditions [12, 13]. Interestingly, Guasch-Vidal et al. [14] demonstrated that, even in the absence of flavonoids, CIAT 899 is able of synthesizing NF in the presence of high concentrations of salt, and the biological activity of these NF was confirmed. Moreover, the activation under salt stress is independent of NodD1 [14]. It has been reported that initial steps of rhizobium-legume symbioses are very sensitive to salt stress. However, the ability to form root nodules on their host legume species under saline conditions has been described for many rhizobia [15]. In general, rhizobial strains use distinct mechanisms for osmotic adaptation under salt stressing conditions during the free-lifestyle, such as the intracellular accumulation of osmolytes and specific ions, modification in cell surface polysaccharides or the synthesis of certain ABC membrane transporters [15, 16]. However, the synthesis of salt-induced NF has been only reported in CIAT 899.

Genome sequencing of CIAT 899 revealed five different *nodD* genes and three different *nodA* genes in the symbiotic plasmid [17]. NodA catalyzes the transfer of the fatty acyl group from an acyl carrier protein to a terminal n-glucosamine residue previously deacetyled by NodB, on the chitin oligomer [18]. The *nodA1* gene is located adjacent to *nodD1*, whose encoded protein seems to be the major regulator of NF synthesis upon induction with flavonoid [19, 20] and together with *nodBC* compose an operon responsible for the synthesis of the NF core. The *nodA2* gene is part of a gene cluster including *hsnT* and *nodFE,* implied in unsaturated fatty acid incorporation into NF molecules and is located close to the *nodD2* gene. Curiously, a previous study [20] showed that, apparently, the activation of the expression of the *nodC* gene under salt stress is lower in a *nodD2* mutant than in both a *nodD1* mutant and the wild-type strain. Finally, *nodA3* is located downstream the *nodD3* gene but no other symbiotic-related genes have been identified in its vicinities [21].

The main objective of this study was to identify genes of CIAT 899 that are differentially expressed in the presence of the *nod*-gene inducer molecules apigenin and salt by RNA-seq analysis. In addition, the promoter regions of the symbiotic genes that were up-regulated in both conditions were studied to determine possible conserved promoter consensus motifs. Our results showed similar patterns of expression for the differentially expressed genes of the symbiotic plasmid replicon in the presence of both apigenin and salt, indicating that the NF synthesis was carried out following the same pathway, independently of the inducer molecule. The biological significance of the CIAT 899 transcriptomic response under salt condition was discussed.

## Results and discussion

### Identification of the differentially expressed genes

To identify differentially expressed genes in the presence of *nod* gene inducers six RNA-seq libraries were generated from CIAT 899 grown in the presence of apigenin (3.7 μM), salt (300 mM) or under control conditions. Two independent biological experiments were carried out for each condition, being the general features of each run shown in Additional file 1. Libraries were sequenced and a range of 54 to 210 million reads were obtained in each condition, indicating that similar amounts of data were generated independently of the growth condition. Three different RNA-seq metrics for quality control, such as GC content, duplicate distribution, and the distribution of respective genetic coordinates, were performed (Additional file 1). Besides, before all subsequent analysis, a normalization of the quantitative data was performed to avoid statistical deviations due to differences in library and genetic sizes [22] (Additional file 1). Data set were validated by *q*RT-PCR (Table 1). In all cases, positive correlation degrees were obtained in fold-change values of the *q*RT-PCR and the RNA-seq data (Additional file 2).

**Table 1:** RNA-seq data validation using *q*RT-PCR. Fold-change values were calculated using the ∆∆Ct method and normalized to the reference gene RNA 16S for 20 differentially expressed genes. HP: gene that codes for a hypothetical protein

Gene name	Locus tag	Nucleotide range	RNA-seq		*q*RT-PCR	
			Apigenin	Salt	Apigenin	Salt
*araC1*	RTCIAT899_CH06050	1238211_1239707	1.81	6.98	1.60	3.49
*rpsL*	RTCIAT899_CH07390	1516428_1516799	−1.61	12.47	3.20	7.11
*araC2*	RTCIAT899_CH14150	2846615_2847376	−2.56	−8.00	1.97	2.04
*y4wE*	RTCIAT899_PB00575	97717_98829	8.58	12.17	15.35	16.68
*y4wF*	RTCIAT899_PB00570	96476_97504	3.20	7.11	12.05	10.04
*nodA2*	RTCIAT899_PB01095	192166_192756	10.30	9.81	13.90	17.00
*hsnT*	RTCIAT899_PB01100	192929_194854	7.27	5.94	25.67	12.50
*nodF*	RTCIAT899_PB01105	194950_195231	13.39	13.43	11.90	13.45
*nodE*	RTCIAT899_PB01110	195232_196440	10.37	11.94	16.74	13.78
*nodA1*	RTCIAT899_PB01300	235667_236257	6.69	12.00	14.18	17.83
*nodB*	RTCIAT899_PB01305	236254_236913	11.4	19.01	8.55	16.30
*nodC*	RTCIAT899_PB01310	236925_238283	6.69	12.00	11.39	15.94
*nodS*	RTCIAT899_PB01315	238201_238917	7.53	12.89	6.03	10.03
*nodU*	RTCIAT899_PB01320	238953_240680	5.39	10.18	15.29	22.86
*nodI*	RTCIAT899_PB01325	240668_241582	6.59	12.48	7.58	11.89
*nodJ*	RTCIAT899_PB01330	241586_242371	4.98	10.15	3.92	15.83
*nodH*	RTCIAT899_PB01340	242956_243705	3.09	7.6	14.80	17.02
HP	RTCIAT899_PB01545	281777_282742	4.27	14.37	8.88	18.11
*nodM*	RTCIAT899_PB02710	502220_504046	2.43	5.85	5.69	12.58
HP	RTCIAT899_PC04980	1059004_1060056	1.32	−4.22	1.14	−5.38

**Fig. 1:**
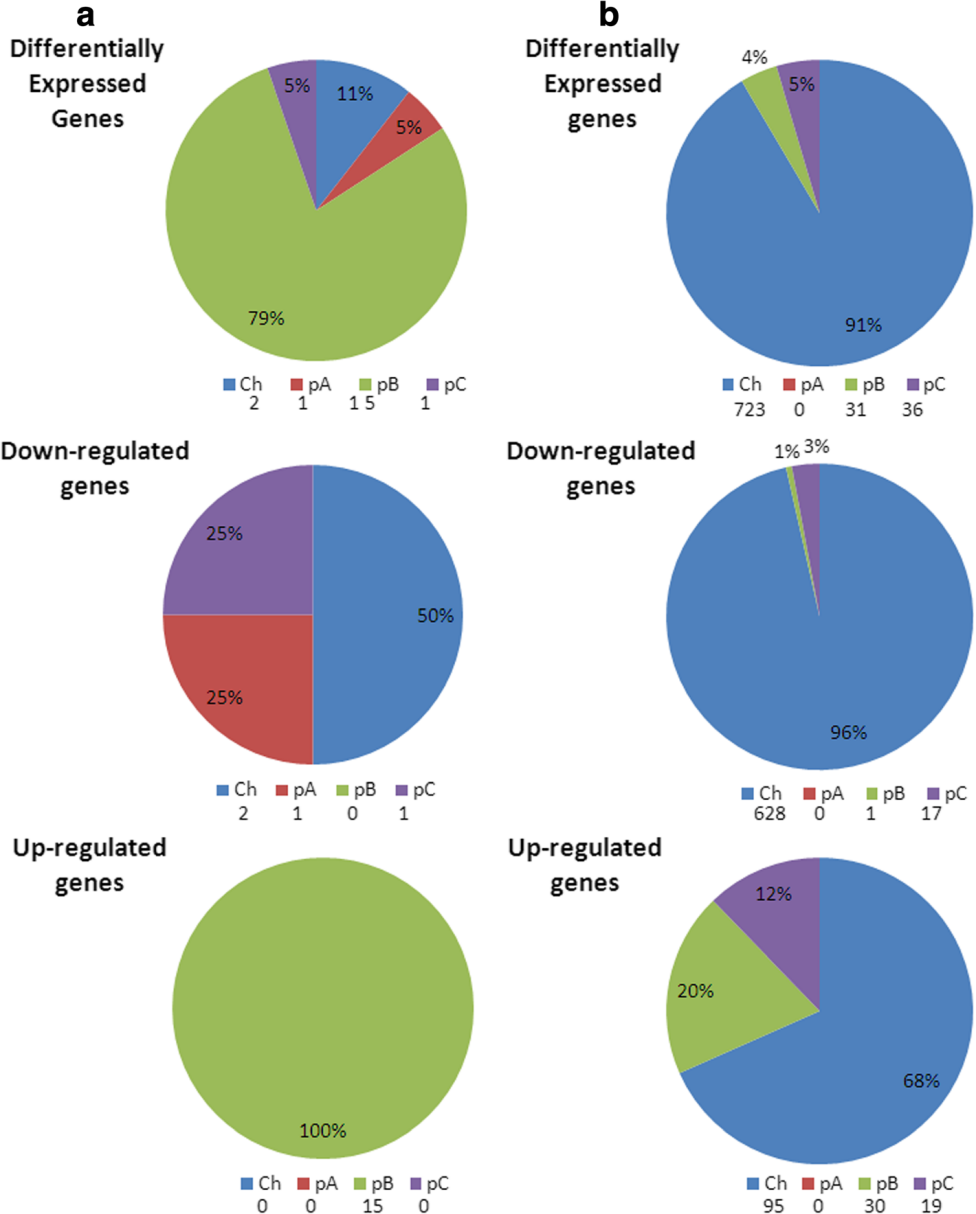
Distribution of differentially expressed genes. Percentage and location of differentially expressed genes (up- and down-regulated) in *R. tropici* CIAT 899 induced with apigenin (**a**) or salt (**b**). Ch: chromosome, pA: pRtrCIAT899a, pB: pRtrCIAT899b, pC: pRtrCIAT899c. The number of differentially expressed genes is indicated under each replicon

*R. tropici* CIAT 899 genome contains 6289 genes distributed among one chromosome (3672 CDS, GeneBank number CP004015.1) and three different plasmids: pRtrCIAT899a/pA (212 CDS, GeneBank number CP004016.1), pRtrCIAT899b/pB/symbiotic plasmid (500 CDS, GeneBank number CP004017.1), and pRtrCIAT899c/pC (1905 CDS, GeneBank number CP004018.1) [17]. Differentially expressed genes in each condition were obtained using the statistical software R. Results revealed 19 differentially expressed genes when the bacterium was grown in the presence of apigenin (0.3 % of the genome) and 790 genes when the bacterium was grown under salt stress condition (12.5 % of the genome). In the first case, 15 genes were up-regulated (78.9 %) and located in the symbiotic plasmid, while the other 4 were down-regulated and distributed in the other replicons: chromosome (2) and plasmids A (1) and C (3) (Fig. 1a). With respect to the cultures supplemented with salt, the majority of genes were down-regulated (646, 81.6 %); only 144 genes were over-expressed representing 18.4 % of the differentially expressed genes (Fig. 1b). Replicon distribution revealed that most of these genes (723; 95 up-regulated and 628 down-regulated) were located in the chromosome, some in plasmid B (31; 30 up-regulated and 1 down-regulated) and the rest in plasmid C (36; 19 up-regulated and 17 down-regulated) (Fig. 1b).

**Fig. 2:**
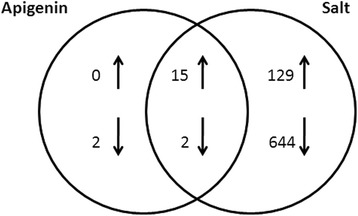
*R. tropici* CIAT 899 responses to apigenin and salt stress. Venn diagram showing the overlapping of differentially expressed genes in the presence of inducer molecules. The arrows indicate the number of up-regulated and down-regulated genes in each treatment, being the number of overlapping intersections indicative of genes expressed in both conditions

Considering all genes, 17 were differentially expressed in both conditions, 15 were up-regulated and located in the symbiotic plasmid and the other two were down-regulated and located in the chromosome (Fig. 2). In addition, many other genes were differentially expressed only under saline conditions. This could be due to a survival strategy when the bacterium faces an abiotic stress. Similar results have been recently reported in *R. etli* CE3 under saline shock conditions, when 6.57 % of the genes were differentially expressed, most of them were located in the chromosome [16].

### Functions associated to responses to *nod* gene-inducing molecules

**Fig. 3:**
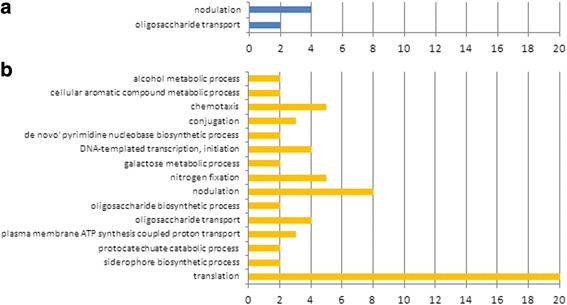
Number of regulated genes from each over-represented biological process (Gene Onthology, GO). Biological processes and number of genes affected in bacterial cultures supplemented with apigenin (yellow) **a** or salt (blue) (**b**). Over-represented functional categories were those with a *p* value in hypergeometrical test inferior to 0.15

CIAT 899 is able to synthesize NF not only upon induction with flavonoid but also under salt stress [14]. To evaluate which functions are intrinsic to each inducing condition, a functional enrichment was carried out to assign the statistically over-represented biological processes (activated or repressed) using data available at the Uniprot database (Gene Ontology, GO). Results are summarized in Additional file 3. As expected, when induced with apigenin, some of the differentially expressed genes were involved in nodulation and oligosaccharide transport (Fig. 3a). The same biological processes were also activated under salt stress. However, the presence of salt also induced many other biological processes such as nitrogen fixation, chemotaxis, carbohydrate metabolism, transcription, translation, conjugation and ATP biosynthesis (Fig. 3b). RNA-seq and proteomics studies performed on other rhizobial strains under saline and heat shock conditions revealed that many of these processes are also being altered [16, 23, 24].

### Apigenin activates the transcription of *nod* gene operons and the synthesis of indole-3-acetic acid

**Fig. 4:**
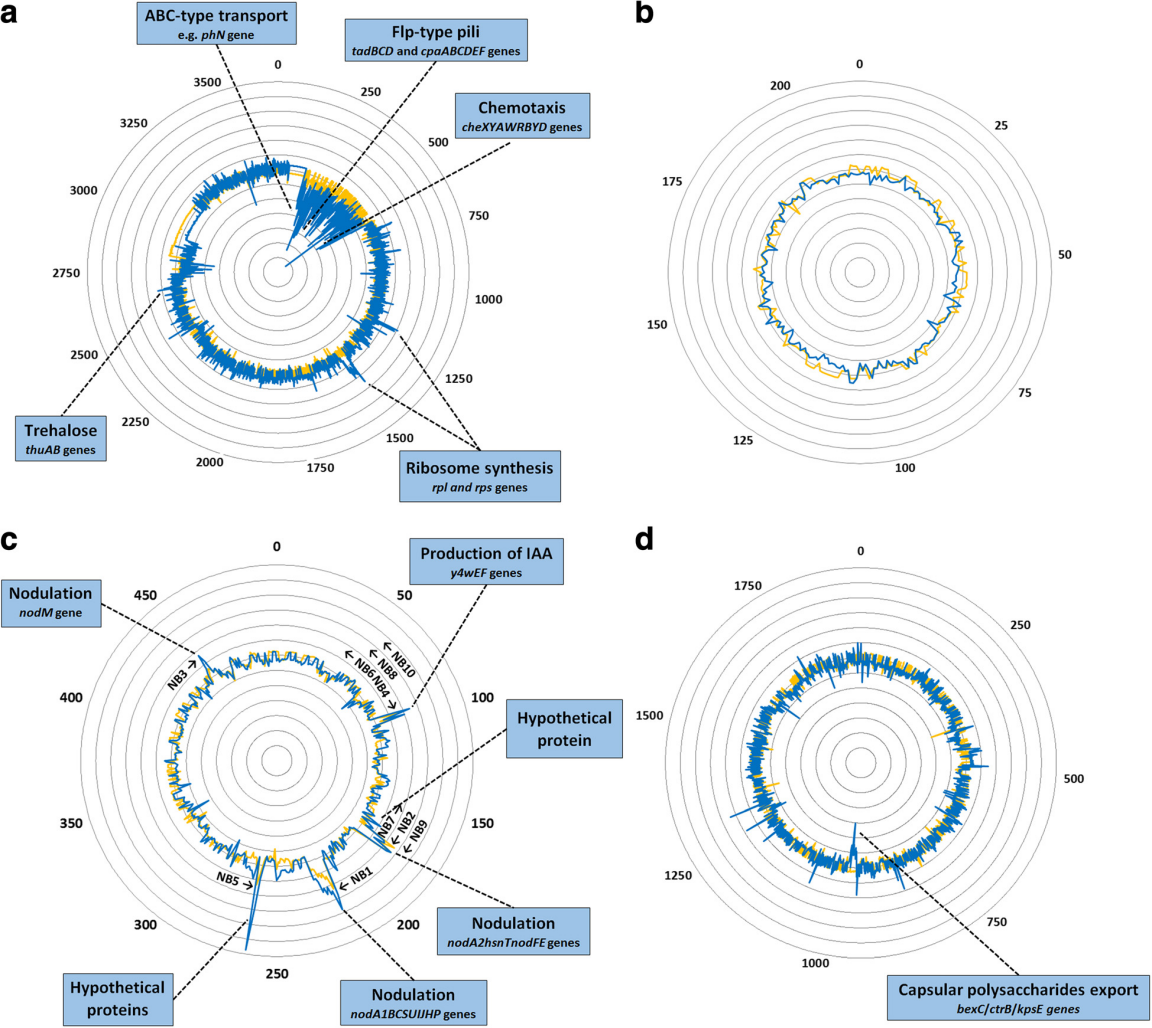
Circular representation of the complete RNA-seq-based transcriptomic data set of apigenin and salt cultures versus standard cultures for all replicons of *R. tropici* CIAT 899. Fold-change values of consecutive genes are represented by linked peaks. Each peak corresponds to one gene, being ordered according to their relative position in the replicon. Yellow peaks: differentially expressed genes in the presence of apigenin. Blue peaks: differentially expressed genes under salt stress. The arrows indicate names and related biological processes of some differentially expressed genes. NB: *nod* box. **a** Chromosome (3.8 Mb), **b** pRtrCIAT899a (0.22 Mb), **c** pRtrCIAT899b (0.55 Mb), **d** pRtrCIAT899c (2.08 Mb)

The CIAT 899 genome harbors five different *nod* genes and three different *nodA* genes in the symbiotic plasmid [17]. Are the *nodA1BC* and the *nodA2FE* operons and the *nodA3* gene of CIAT 899 activated by apigenin? Transcriptomic analysis revealed that four sets of genes were significantly up-regulated in the symbiotic plasmid under this condition. Two of them corresponded to the *nodA1* and *nodA2* operons, the third, located adjacent to the *nodD5* gene, was involved in the synthesis of the phytohormone indole-3-acetic acid (IAA) [21], and the last set of genes coded for proteins with unknown functions (Fig. 4, Additional file 4). Therefore, at least two main biological processes were activated in CIAT 899 when induced with apigenin: the synthesis of NF (*nodA1BCSUIJHPQ1Q2* and *nodA2hsnTnodFE* genes) and the production of IAA (*y4wEF* genes). Of special interest is the elucidation of the symbiotic role of the set of genes with unknown function activated with apigenin (RTCIAT899_PB01550 and RTCIAT899_ PB01545). In summary, these results indicate that when induced with apigenin, CIAT 899 is able to synthesize NF that will induce root nodule primordia formation, as well as phytohormones that will favor root development. This is consistent with a recent study in which an increase in the production of IAA is reported when CIAT 899 is grown in cultures supplemented with both apigenin and salt [20]. A similar regulation cascade involving NodD1 and flavonoids has been reported in *Sinorhizobium fredii* NGR234 for the synthesis of IAA [25].

### Common responses: synthesis of nodulation molecules and identification of *nod* boxes

As previously mentioned in the introduction, the capacity to nodulate under salt stress conditions has been described for many rhizobial strains [15]. However, the salt-regulated production of NF has been only reported for *R. tropici* CIAT 899. Are the CIAT 899 genetic pathways to synthesize NF under salt stress similar to those described for apigenin? The RNA-seq analysis shown in this work indicated that although the pattern of symbiotic gene activation in the presence of salt was similar to that obtained with apigenin (Fig. 4, Additional files 4 and 5), slightly higher expressions of *nod* genes, with an emphasis on the *nodA1* operon, was observed when the bacterium was induced with salt (Table 2). Moreover, under salt stress *nodM* and a gene that codes for a flavine mononucleotide reductase (RTCIAT899_PB02705) were significantly up-regulated. Although the up-regulation of *nodM* was not detected in the RNA-seq of CIAT 899 grown in the presence of apigenin, it was detected in the *q*RT-PCR analysis (5.69 fold-change) (Table 1).

**Table 2:** Fold-change expression values of the *R. tropici* CIAT 899 *nod* genes on the RNA-seq analysis. Fold-change values followed by an asterisk (*) are significantly over-expressed

Gene name/Locus tag	Apigenin	Salt
*nodD1*/RTCIAT899_PB01295	1.23	1.04
*nodD2*/RTCIAT899_PB01070	−1.25	3.01
*nodD3*/RTCIAT899_PB00640	−1.21	2.1
*nodD4*/RTCIAT899_PB01560	1.09	1.16
*nodD5*/RTCIAT899_PB00560	−1.12	−1.09
*nodA1*/RTCIAT899_PB01300	8.73*	13.66*
*nodB*/RTCIAT899_PB01305	11.4*	19.01*
*nodC*/RTCIAT899_PB01310	6.69*	12*
*nodS*/RTCIAT899_PB01315	7.53*	12.89*
*nodU*/RTCIAT899_PB01320	5.39*	10.18*
*nodI*/RTCIAT899_PB01325	6.59*	12.48*
*nodJ*/RTCIAT899_PB01330	4.98*	10.15*
*nodH*/RTCIAT899_PB01340	3.09	7.6*
*nodP*/RTCIAT899_PB01345	2.78	6.08*
*nodQ1*/RTCIAT899_PB01350	3.25	6.39*
*nodQ2*/RTCIAT899_PB01355	1.28	2.54
*nodA2*/RTCIAT899_PB01095	10.3*	9.81*
*hsnT*/RTCIAT899_PB01100	7.27*	5.94*
*nodF*/RTCIAT899_PB01105	13.39*	13.43*
*nodE*/RTCIAT899_PB01110	10.37*	11.94*
*nodA3*/RTCIAT899_PB00645	1.15	3.36
*nodM*/RTCIAT899_PB02710	2.43	5.85*

**Fig. 5:**
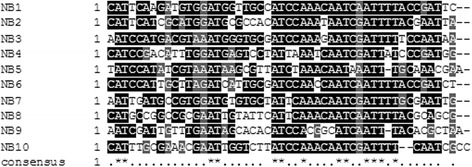
Alignment of NB sequences from *R. tropici* CIAT 899. Software fuzznuc of the EMBOSS package was used to identify *nod* box-like sequences. Hits were selected based on their conservation to previously identified NB sequences. Searching of these consensus sequences was carried out on the whole genome of *R. tropici* CIAT 899. DNA sequences were aligned using the ClustalW program and manipulated with Boxshade at EMBnet. Dark and gray boxes indicate identical and similar nucleotides, respectively. Promoter consensus motifs are marked with an asterisk on the consensus line

All these results indicate that the transcription of the CIAT 899 *nod* genes seems to respond similarly to the presence of either apigenin or salt (Table 2). The question that remains unclear is whether the responses observed follow the common NodD activation pathway. To answer this question, an *in silico* analysis to detect promoter motifs was conducted using consensus sequences of *nod* boxes (NB) present in other related rhizobial strains. Thus, ten potential NB were identified, all sharing the AT-N_11_-AT-N_7_-AT-N_2_-A-N_4_-AT-N_2_-ATT-N-T sequence consensus (Fig. 5). Four of these NB were situated upstream of the four different sets of genes up-regulated under both inducing conditions, namely, the operons *nodA1BCSUIJHPQ1Q2* (NB1), *nodA2hsnTnodFE* (NB2), *y4wEF* operon (NB4) and the *nodM* gene (NB3) (Table 3, Additional files 4 and 5). Interestingly, two of the genes previously identified as up-regulated with apigenin (RTCIAT899_PB01550 and RTCIAT899_ PB01545) were also induced with salt. The expression of one of these genes (RTCIAT899_PB01545) was validated by *q*RT-PCR in the presence of both apigenin (8.88-fold) and salt (18.11-fold) (Table 1). This gene expression, combined with the fact that both genes were located downstream NB5, indicate that these hypothetical proteins could be playing a role in the symbiotic process (Table 3, Additional files 4 and 5). Finally, NB7 was located upstream a gene (RTCIAT899_PB01055) up-regulated with salt, but not with apigenin (Table 3, Additional file 5).

**Table 3:** Up-regulation of the *R. tropici* CIAT 899 pRtCIAT899b genes located downstream *nod* boxes. Transcriptional activation (4-fold induction with respect to control cultures) of several *nod* box controlled operons was demonstrated by RNA-seq data in the presence of both inducer molecules. HP: gene that codes for a hypothetical protein

NB (Distance)	Cds number/Locus tag/Gene name	Putative function	Fold change apigenin	Fold change salt
1 (270 pb)	cds216 to 227/RTCIAT899_ PB01300 to RTCIAT899_ PB01345/*nodABCSUIJHPQ1Q2*	NF production	8.73 to 1.28	13.66 to 2.54
2 (250 pb)	cds177 to 180/RTCIAT899_ PB01095 to RTCIAT899_ PB01110/*nodA2hsnTnodFE*	NF production	10.3 to 10.37	9.81 to 11.94
3 (180 pb)	cds450 to 449/RTCIAT899_ PB02710 to RTCIAT899_ PB02705/*nodM*	NF production	2.43 to 3.18	5.85 to 8.49
4 (680 pb)	cds95 to 93/RTCIAT899_PB00575 to RTCIAT899_PB00565/*y4wEF*	Synthesis of IAA	8.57 to 1.63	12.17 to 3.27
5 (38 pb)	cds263 to 262/RTCIAT899_PB01550 to RTCIAT899_PB01545/HP	Unknown	6.75 to 4.26	28.65 to 14.37
6 (70 pb)	cds61 to 62/RTCIAT899_PB00370 to RTCIAT899_PB00375/HP	Unknown	1.80 to 2.11	2.17 to 1.64
7 (191 pb)	cds170/RTCIAT899_PB01055/HP	Unknown	1.18	5.68
8 (470 pb)	cds81/RTCIAT899_PB00495/HP	Unknown	−1.05	2.58
9 (979 pb)	cds173/RTCIAT899_PB01570/*nodD2*	Transcriptional regulation	−1.25	3.01
10 (292 pb)	cds89/RTCIAT899_PB00540/HP^a^	Unknown	1.13	−1.44

^a^The transcriptional orientation of this gene is opposite to the NB10 sequence orientation

Altogether, these results suggest that when CIAT 899 was induced either by apigenin or salt, genes related to the synthesis of NF and IAA were activated through the recognition of specific *nod* boxes (Table 2). In *S. fredii* NGR234, nineteen *nod* boxes have been identified and eighteen of them were inducible with flavonoids via NodD1. In addition, *S. fredii* NGR234 carries two copies of the *nodD* gene and four of these *nod* boxes are controlled by NodD2 [26].

### Salt stress response: a pathway towards symbiosis?

Chromosomal RNA-seq analysis showed, under salt stress, an up-regulation of genes whose products are implied in the formation of ribosomes (both 30 and 50S ribosomal proteins coded by the *rpl* and *rps* genes, respectively) and in the utilization of the disaccharide trehalose (*thuAB* genes), an osmotic stabilizer [27, 28]. Instead, down-regulation was detected in genes that encode proteins involved in chemotaxis (*cheXYAWRBYD* genes) [29], formation of Flp-type *pili* (type IVb protein secretion system; *tadBCD* and *cpaABCDEF* genes) [30], or ABC-type transport (several genes: e.g. *phn* genes). In addition, a set of genes located in pRtrCIAT899c and involved in the export of capsular polysaccharide across the inner membrane (genes of the family *bexC/ctrB/kpsE*) was also down-regulated (Fig. 4, Additional file 5) [31].

**Fig. 6:**
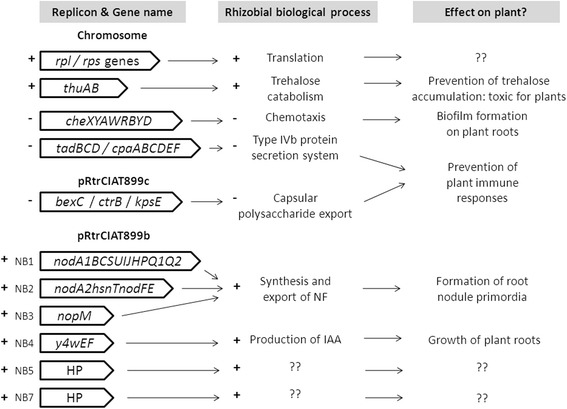
Model of the *Rhizobium tropici* CIAT 899 transcriptomic response under salt stress. RNA-seq studies indicate that in the presence of high concentration of salt CIAT 899 increase the transcription of nodulation genes and the synthesis of NF. Transcriptional activation (+) or inhibition (−) of other biological processes associated with the presence of salt supports the hypothesis that salt-dependent production of NF could be a special *R. tropici* CIAT 899 strategy to ensure nodulation under salt stress conditions. HP: gene that codes for a hypothetical protein

Therefore, the global response of CIAT 899 to salt stress indicates that the strain drastically reduces the uptake of molecules and the energy consumption, reducing the ATP-dependent transport of the ABC-type permeases. However, under saline shock conditions *R. etli* CE3 over-expresses genes that code for many ABC transporters and increases the transcription levels of genes related to the biosynthesis of trehalose [16]. Nevertheless, in CIAT 899 an up-regulation of genes involved in the degradation of trehalose was detected. The explanation could be that this osmolyte tends to accumulate during symbiosis, but it is toxic to plants. To solve this problem, bacteria would produce trehalose-degrading enzymes [32]. These observations in CIAT 899, combined with the production of NF and IAA under salt stress, make us formulate the next question: could the promotion of the symbiosis represent a strategy of CIAT 899 to ensure nodulation under this abiotic stress condition? To shed light on this question we have analyzed the changes in biological processes under saline conditions. First, we observed an enhancement in ribosomal synthesis, which could be related to a translation increase due to an increase in the transcription of nodulation genes (a stronger *nod* gene up-regulation was detected in salt stress conditions). Second, general protein secretion systems and surface polysaccharides seem to play an important role in bacterial fitness under stressing environmental conditions, such as high salinity or temperature [16, 23, 24]. However, in CIAT 899 we observed inhibition under salt stress. Remarkably, both cellular components can also be considered microbe-associated molecular patterns (MAMP), which trigger the plant immune system [33]. Other rhizobial strains in the presence of inducer flavonoids modify their surface molecules to avoid plant immune responses, thereby promoting nodulation [34]. Finally, when colonizing legume roots many rhizobia form micro-colonies or biofilms. In some of these rhizobia common *nod* genes are required for the development of these structures, since the biofilm matrix is composed in part by NF [35, 36]. Thus, in CIAT 899, the synthesis of NF and the down-regulation of chemotaxis genes detected under saline conditions could be related to the formation of a symbiotic biofilm. In summary (Fig. 6), these results suggest that in the presence of salt CIAT 899 could strategically increase the transcription of nodulation genes and the synthesis of NF to increase the chances to establish symbiosis even under abiotic stressing conditions.

## Conclusions

Our work shows that in the presence of the nodulation inducing molecules apigenin (3.7 μM) and salt (300 mM), *R. tropici* CIAT 899 increases the transcription of the same set of genes (slightly higher expression upon salt treatment), whose encoded proteins are involved in the synthesis of symbiotic molecules. Besides, the salt-dependent production of these symbiotic molecules could be a CIAT 899 strategy to ensure nodulation under salt stress.

## Methods

### Culture conditions and RNA extraction

*R. tropici* CIAT 899 was grown for 72 h at 28 °C on tryptone yeast (TY) medium [37], supplemented with apigenin 3.7 μM or NaCl 300 mM when necessary (induction of the synthesis of NF) [14, 20]. Total RNA was isolated using a High Pure RNA Isolation Kit (Roche), according to the manufacturer’s instructions. Verification of the amount and quality of total RNA samples was carried out using a Nanodrop 1000 spectrophotometer (Thermo Scientific) and a Qubit 2.0 Fluorometer (Invitrogen). Two independent total RNA extractions were obtained for each condition.

### Quantitative reverse transcription PCR

Result obtained in the RNA-seq analysis were validated by quantitative reverse transcription PCR (*q*RT-PCR) of 20 selected genes, which represented differentially and non-differentially expressed genes in the presence of apigenin and salt. Total RNA was isolated using a High Pure RNA Isolation Kit (Roche) and RNAase Free DNA Set (Qiagen), according to the manufacturer’s instructions. This (DNA-free) RNA was reverse transcribed into cDNA using a QuantiTec Reverse Transcription Kit (Qiagen). Quantitative PCR was performed using a LightCycler 480 (Roche) with the following conditions: 95 °C, 10 min; 95 °C, 30 s; 50 °C, 30 s; 72 °C, 20 s; forty cycles, followed by the melting curve profile from 60 to 95 °C to verify the specificity of the reaction. The *R. tropici* CIAT 899 16S rRNA gene was used as an internal control to normalize gene expression. The fold-changes of two biological samples with three technical replicates of each condition were obtained using the ∆∆C_t_ method [38]. Selected genes and primers are listed in Additional file 2.

### RNA sequencing

Ribosomal RNA was depleted using a MICROB Express Bacterial mRNA Purification kit (Ambion), following the manufacturer’s protocol. Integrity and quality of the ribosomal depleted RNA was checked with Agilent Bioanalyzer 2100 (Agilent Technologies). RNA sequencing was carried out by Sistemas Genómicos (https://www.sistemasgenomicos.com/web_sg/) with the Next Generation Sequence (NGS) platform Illumina using the Illumina HiSeq 2000 sequencing instrument (Illumina). Ribosomal-depleted samples were used to generate whole transcriptome libraries following the manufacturer's recommendations for sequencing on this NGS platform. Amplified cDNA quality was analyzed by the Bioanalyzer 2100 DNA 1000 kit (Agilent Technologies) and quantified using the Qubit 2.0 Fluorometer (Invitrogen).

### Mapping of the RNA-seq data

The initial whole transcriptome paired-end reads obtained from sequencing were mapped against the latest version of the *R. tropici* CIAT 899 genome (http://www.ncbi.nlm.nih.gov/genome/?term=Rhizobium_tropici_CIAT_899) using the the Life Technologies mapping algorithm version 1.3 (http://www.lifetechnologies.com/). Low-quality reads were eliminated using Picard Tools software version 1.83, remaining only high quality reads.

### Assessment of differentially expressed genes

Gene prediction was estimated using the cufflinks method [39] and the expression levels were calculated using the htseq software, version 0.5.4p3 [40]. This method eliminates multimapped reads, considering only unique reads for the gene expression estimation. The edge method version 3.2.4 was applied for differential expression analysis among conditions [41]. This method uses a Poisson model to estimate the variance of the RNA-seq data for differential expressions, and relies on different normalized processes based on depth global samples, CG composition and length of genes. Differentially expressed genes were established in those genes with a fold-change lower or higher than−4 or 4, respectively, with a *p* value adjust to 0.7.

### Functional categorization of genes

In order to assign the statistical over-represented functional categories in the presence of both *nod* gene-inducing molecules, an enrichment functional study was performed. Thus, genes were annotated using Uniprot databases and a hypergeometrical test using all genes as background and differential gene expression as interesting group of genes was applied [42]. This statistical test calculates the statistical significance using *p* value [43], being in this case evaluated the significance of functional categories. Those functional categories (biological processes) with a *p* value inferior to 0.15 were considered over-represented.

### Consensus motifs

The program fuzznuc of the EMBOSS package was used to identify *nod* box-like sequences. Hits were selected based on their conservation to known NB sequences [44, 45]. Thus, the search pattern used was at[ct][cg][ag]n(5)[tc][ga][ga]atn(7)at[ct]caaacaatc[ga]attttncn(2)at, allowing a maximum of 3 mismatches. Searching of these consensus sequences was carried out on the whole genome of CIAT 899. Alignment of the NB sequences was performed using ClustalW at EMBnet.

### RNA-seq data accession number

The RNA-seq data discussed in this publication have been deposited in the Sequence Read Archive of NCBI under the accession number SRP067561.

## Additional files

Additional file 1: General features of the total sequenced and mapped reads. Reads were mapped using a Bayesian inference using Cufflinks v2.11 software. Worst quality reads were removed by means of Picard Tools (See the Material and Methods section) (PDF 185 kb) Additional file 2: Correlation degrees between RNA-Seq and *q*RT-PCR experiments. *q*RT-PCR and RNA-seq fold-change values of 20 selected genes were represented in a graphic to obtain the correlation degrees in both conditions. Genes and primer sequences are shown (XLS 38 kb) Additional file 3: Functions (Gene Onthology, GO) associated to *R. tropici* CIAT 899 responses to apigenin and salt*.* A hypergeometric test performed in the platform R (See the Material and Methods section) was carried out to assign the statistical over-represented functional categories in presence of both *nod* gene inducing molecules (XLSX 11 kb) Additional file 4: Whole genome differential expression in *R. tropici* CIAT 899 cultures supplemented with apigenin. Python and R were used to study differential expression among sample groups. Algorism proposed by DESeq2 was used for gene differential expression, taking as a dispersion model a negative binomial distribution. Differentially expressed genes were established in those genes with a fold change lower or higher than−4 or 4, respectively (See the Material and Methods section). Fold-change values are represented by bars. Each bar corresponds to one gene and they are ordered according to their relative position in each replicon. Blue bars: genes not differentially expressed. Yellow bars: differentially expressed genes. The arrows indicate names of some differentially expressed genes. Ch: chromosome, pC: pRtrCIAT899c, pB: pRtrCIAT899b, pA: pRtrCIAT899a (XLSX 750 kb) Additional file 5: Whole genome differential expression in *R. tropici* CIAT 899 cultures supplemented with salt. Python and R were used to study differential expression among sample groups. Algorism proposed by DESeq2 was used for gene differential expression, taking as a dispersion model a negative binomial distribution. Differentially expressed genes were established in those genes with a fold change lower or higher than−4 or 4, respectively (See the Material and Methods section). Fold change values are represented by bars. Each bar corresponds to one gene and they are ordered according to their relative position in each replicon. Blue bars: genes not differentially expressed. Yellow bars: differentially expressed genes. The arrows indicate names of some differentially expressed genes. Ch: chromosome, pC: pRtrCIAT899c, pB: pRtrCIAT899b, pA: pRtrCIAT899a (XLSX 654 kb)
