# Supplementary material for: RNA-seq analysis of the Rhizobium tropici CIAT 899 transcriptome shows similarities in the activation patterns of symbiotic genes in the presence of apigenin and salt
Source: BMC Genomics. 2016 Mar 8;17:198. doi: 10.1186/s12864-016-2543-3 (PMC4782375; doi:10.1186/s12864-016-2543-3)

Supplementary file 1. General features of the total sequenced and mapped reads.

Total Number of Reads: Reads were mapped using a Bayesian inference using Cufflinks v2.11 software. Worst quality reads were removed by means of Picard Tools.

Sample condition	#Mapped Reads
CIAT_1	176338004
CIAT_2	65464444
CIAT_APIG_1	210875582
CIAT_APIG_2	67786472
CIAT_SALT_1	172542528
CIAT_SALT_2	54981832

Two biological and independent experiments were carried out for each condition.

Quality Read Controls: Three different controls were performed to ensure the quality of reads: GC content, duplicate distribution and distribution respect genetic coordinates.

- *GC content*: distribution of GC content on mapped reads. A normal distribution around 45-55% is expected.

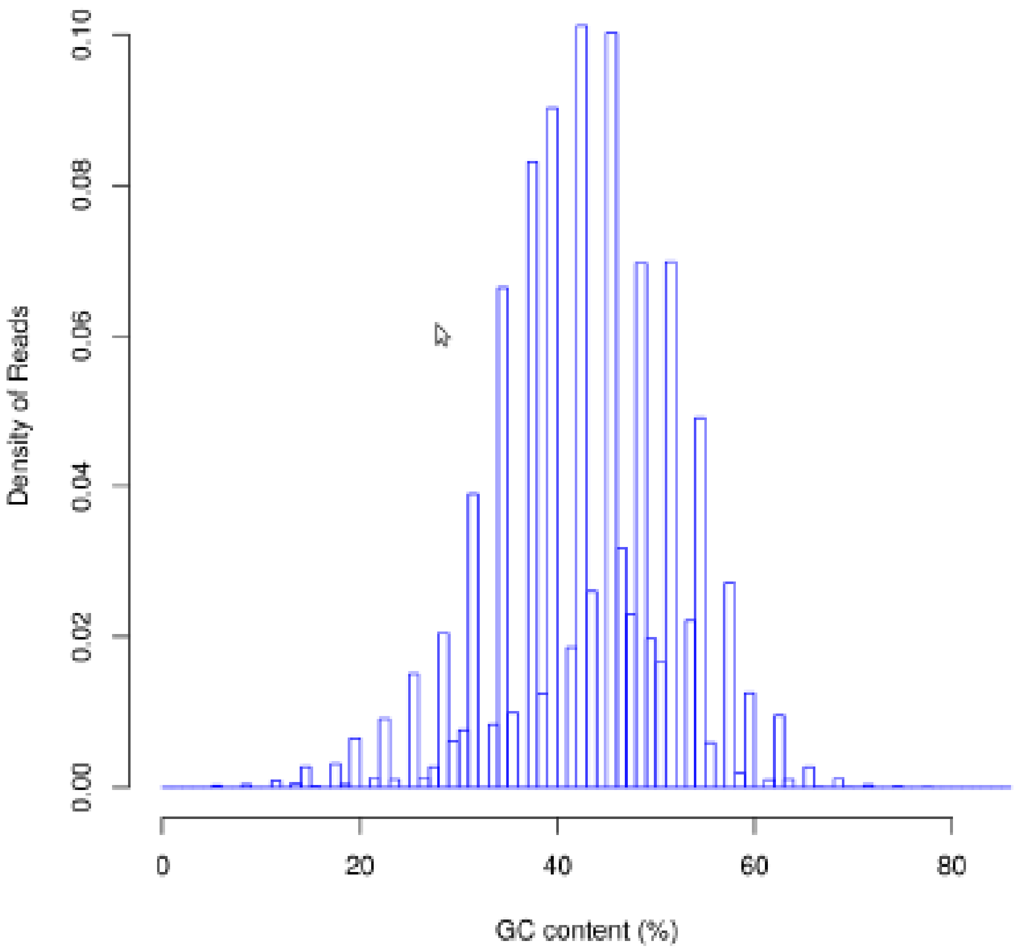

- *Duplicate distribution*: common distribution of duplicates in a RNA-Seq experiment shows a small number of reads with high levels of duplicates and a high number of reads with low levels of duplicates. All samples presented optimal values of duplicate distributions.

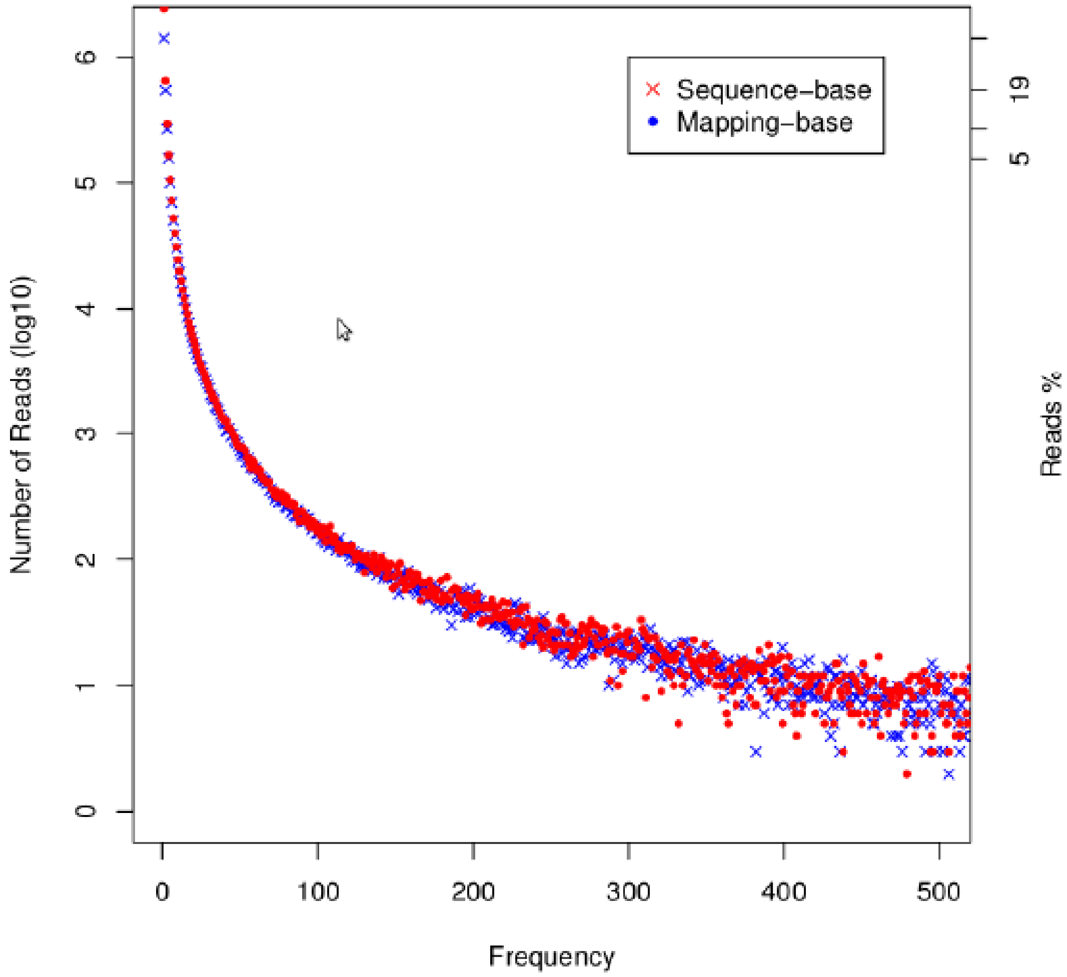

- *Distribution respect genetic coordinates*: centralization around 50 percentile of gene body is expected in high quality samples. Read concentration in both ends could be indicative of RNA degradation.

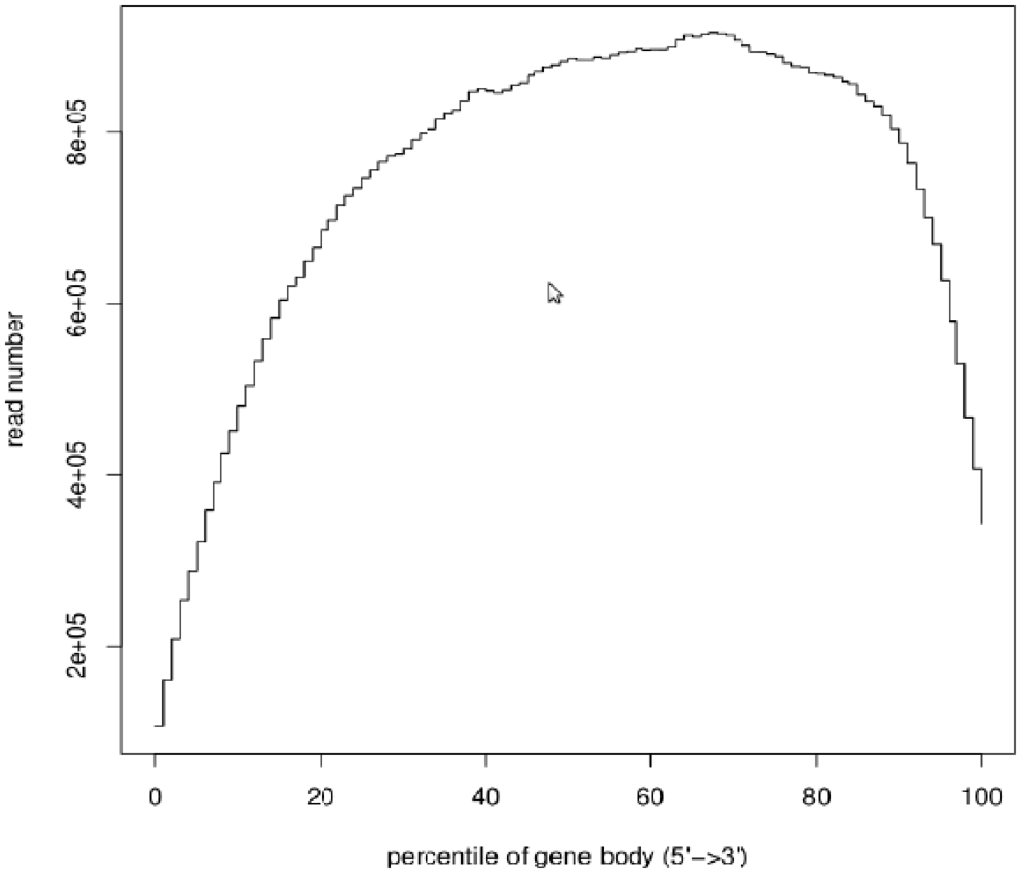

Normalization: normalization is needed to avoid statistical deviations due to differences in library sizes.

Number of reads per gene for control and apigenin samples **before** normalization.

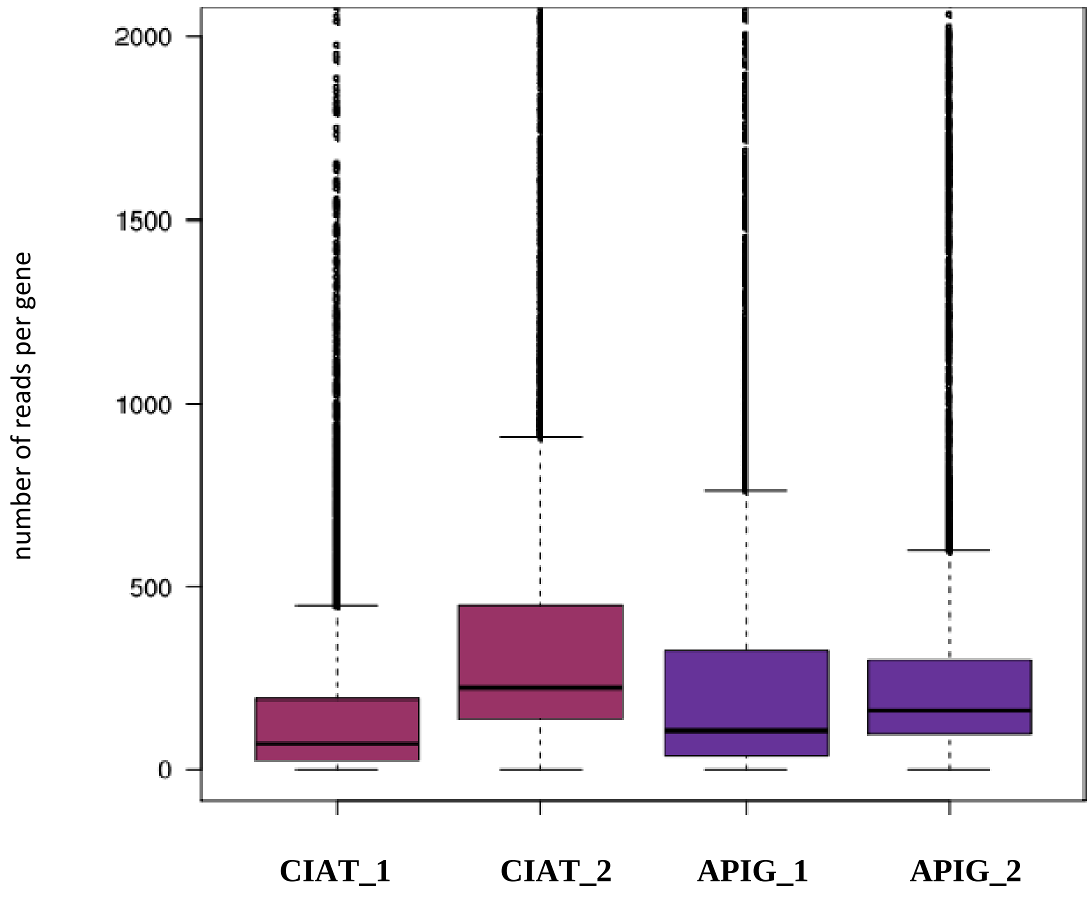

Number of reads per gene for control and salt samples **before** normalization.

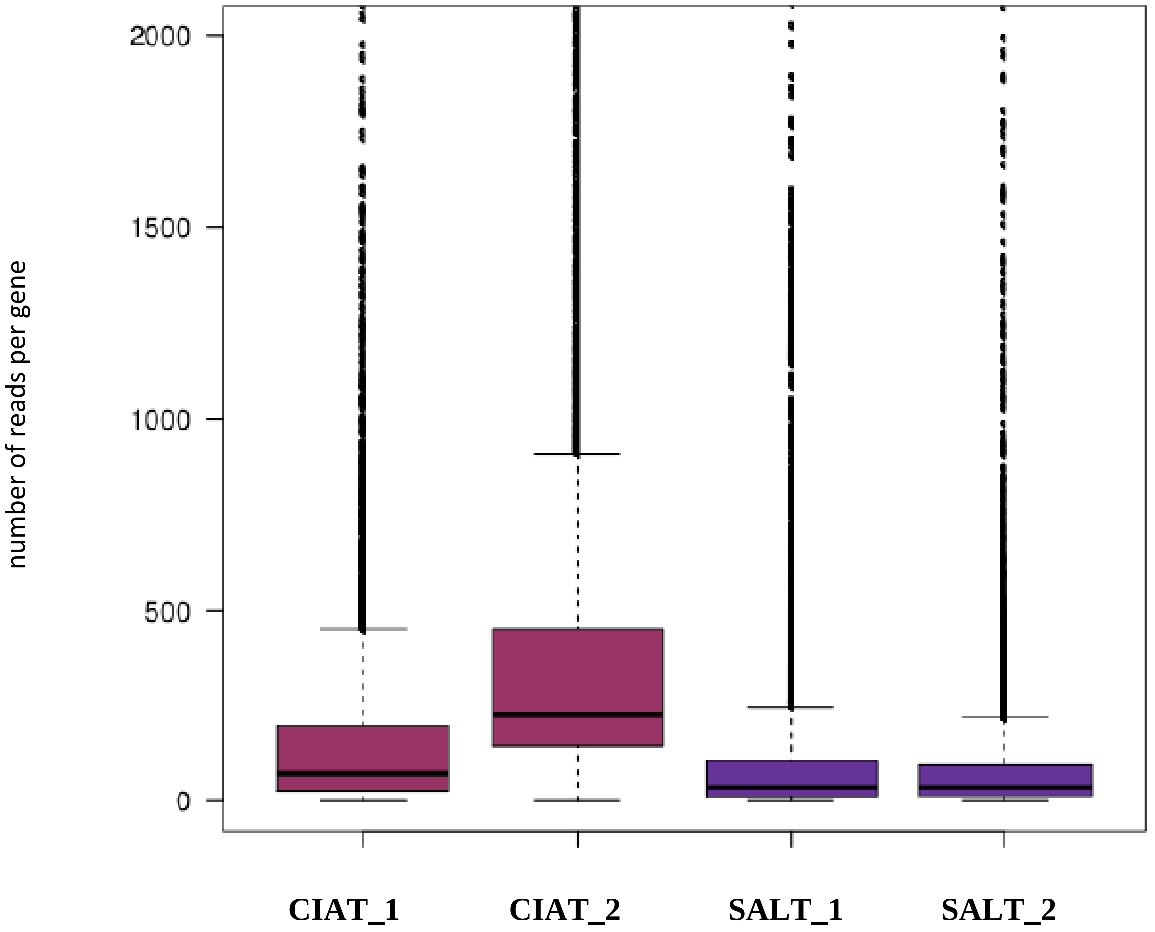

Number of reads per gene for control and apigenin samples **after** normalization.

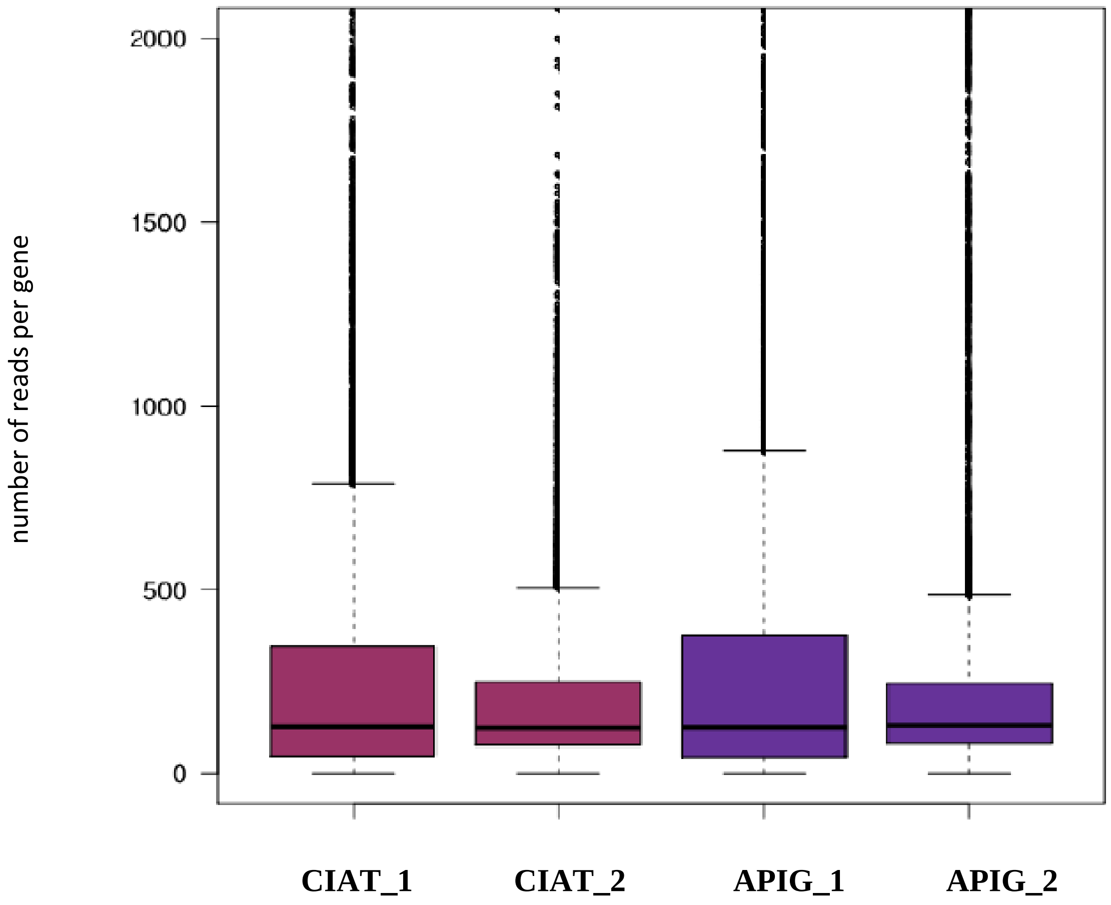

Number of reads per gene for control and salt samples **after** normalization.

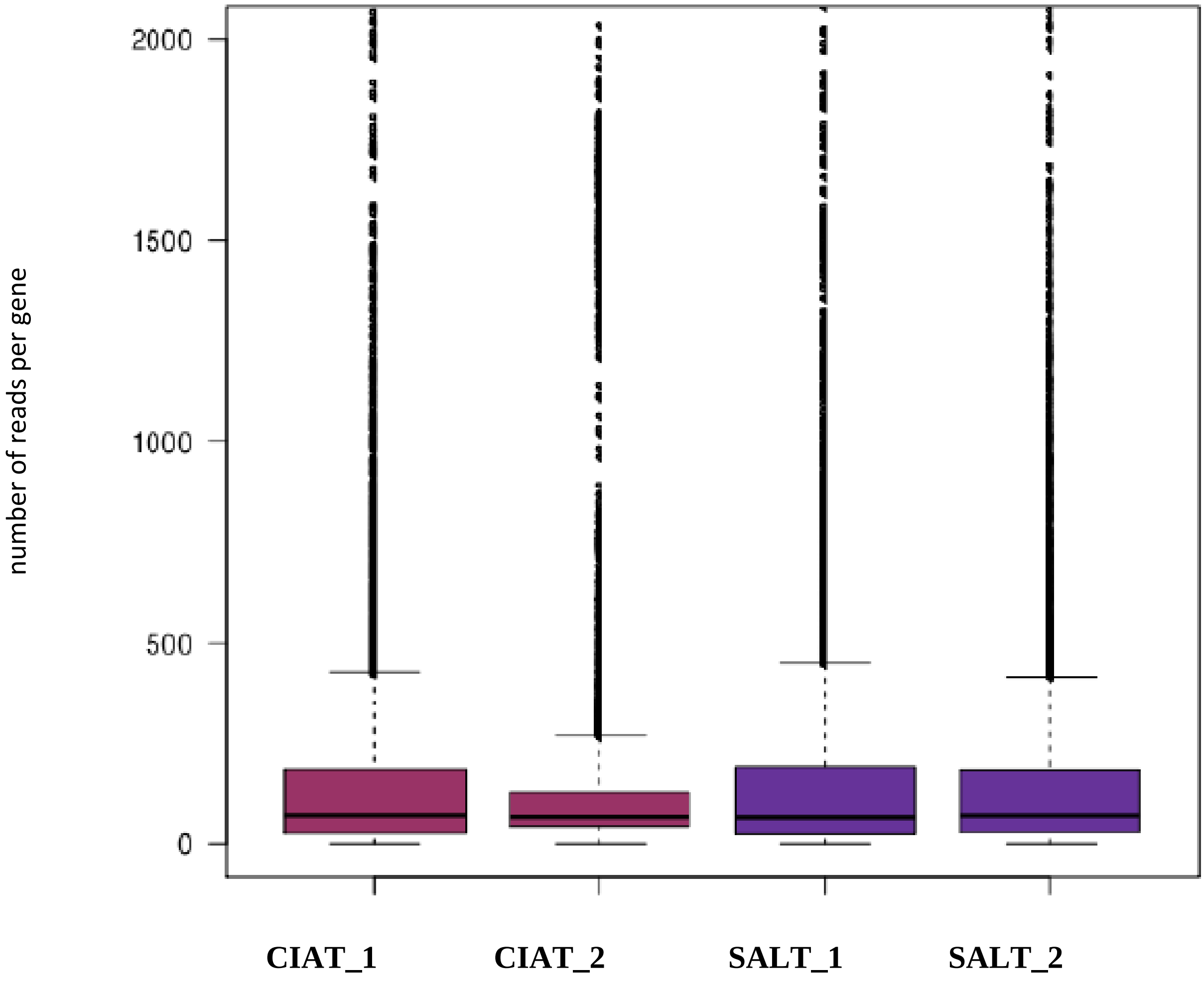

Supplement: Additional file 1: — General features of the total sequenced and mapped reads. Reads were mapped using a Bayesian inference using Cufflinks v2.11 software. Worst quality reads were removed by means of Picard Tools (See the Material and Methods section) (PDF 185 kb) [file 12864_2016_2543_MOESM1_ESM.pdf]
